# Structural and functional alterations in the contralateral hemisphere following pediatric intracranial surgery: a pilot longitudinal neuroimaging study

**DOI:** 10.3389/fnhum.2025.1568945

**Published:** 2025-03-07

**Authors:** Na Yan, Bohan Hu, Huina Zhai, Xu Han, Cuiling Hu, Xueyi Guan, Jian Gong

**Affiliations:** ^1^Department of Neurology, Peking University Shougang Hospital, Beijing, China; ^2^Department of Pediatric Neurosurgery, Beijing Tiantan Hospital, Capital Medical University, Beijing, China; ^3^Beijing Neurosurgical Institute, Capital Medical University, Beijing, China; ^4^Institute of Artificial Intelligence, Hefei Comprehensive National Science Center, Hefei, China; ^5^Beijing RIMAG Medical Imaging Center, Beijing, China; ^6^Department of Radiology, Songjiang Hospital, Songjiang Research Institute, Shanghai Jiao Tong University School of Medicine, Shanghai, China

**Keywords:** pediatric neurosurgery, brain tumor, longitudinal trajectory, cognition, fMRI

## Abstract

**Background:**

Intracranial space-occupying lesions (ISOLs) are common pediatric conditions. Recent therapeutic advances have significantly improved survival rates, necessitating increased attention to post-operative cognitive outcomes, which are crucial determinants of patients’ quality of life.

**Objective:**

While previous studies have predominantly focused on short-term post-operative changes, this study aimed to investigate longitudinal changes in cognition, brain structure, and function of the contralateral hemisphere following pediatric neurosurgery.

**Methods:**

Nineteen pediatric patients with ISOLs were enrolled in a paired design study. Cognitive assessments, structural imaging, and functional imaging data were collected at three time points: pre-operation, first post-operative follow-up (mean 75 days pre-operation), and second post-operative follow-up (mean 316 days pre-operation). Relevant metrics were computed and compared across time points.

**Results:**

The majority of cognitive domains exhibited a gradual longitudinal improvement trajectory, with three domains showing significant enhancement at the second follow-up compared to preoperative baseline: cognitive flexibility (*t* = 4.201, *p* = 0.001), executive function (*t* = 3.478, *p* = 0.003), and social accuracy (*t* = 3.248, *p* = 0.004). The contralesional hemisphere demonstrated alterations primarily characterized by gray matter density reduction, progressing from subcortical structures (first follow-up: thalamus, peak intensity = −7.54, cluster *p* < 0.016) to cortical regions (second follow-up compared to previous follow-up: superior frontal gyrus, peak intensity = −7.80, cluster *p* < 0.016), followed by a subsequent increase in brain activity power of smaller magnitude (second follow-up: medial superior frontal gyrus, amplitude of low frequency fluctuation, peak intensity = 5.96, cluster *p* < 0.016). Correlation analysis suggests that there is an association between changes in brain structure and alterations in cognitive function (*r* = −0.53, *p* = 0.019).

**Conclusion:**

Our findings suggest that post-craniotomy structural and functional brain changes in children follow a subcortical-to-cortical trajectory, with structural alterations (decreased gray matter density) preceding functional activation. This process demonstrates progressive and cumulative characteristics. These modifications appear to correlate with cognitive function recovery and may represent potential mechanisms underlying spontaneous cognitive rehabilitation in pediatric patients post-surgery. Cautiously interpreted, the deeper neuroplastic mechanisms underlying these changes might involve synaptic pruning-like processes induced by external perturbation.

## 1 Introduction

Intracranial space-occupying lesions (ISOLs) represent one of the prevalent pathological conditions in pediatric populations. Brain tumors constitute the most common solid neoplasms in children (Subramanian and Ahmad, 2021). Central nervous system tumors account for 20% of pediatric malignancies, with an incidence rate second only to leukemia (Patel et al., 2014). Surgical intervention remains the primary therapeutic approach for ISOLs, including brain tumors. Over recent decades, technological advancements have contributed to improved prognoses for numerous pediatric brain tumors (Ostrom et al., 2021). Across most histological subtypes, children and adolescents typically demonstrate superior survival outcomes compared to their adult counterparts (Ostrom et al., 2021). This disparity may be attributed to two principal factors: the remarkably high prevalence of low-grade brain tumors in children (Sturm et al., 2017) and their enhanced neuroplasticity (Iuvone et al., 2011), which confers greater resilience to surgical interventions and other therapeutic modalities. Given these circumstances, there is an urgent need to investigate the underlying mechanisms of post-operative cognitive changes in pediatric patients. Such research would significantly contribute to our understanding of cognitive rehabilitation in children and potentially reduce post-operative disability rates.

T1-weighted imaging provides detailed anatomical visualization for structural studies, while resting-state functional magnetic resonance imaging (rs-fMRI) captures spontaneous fluctuations in blood oxygen level-dependent (BOLD) signals (Lee et al., 2013; Smitha et al., 2017), offering insights into intrinsic brain activity (Lv et al., 2018).

In recent years, neuroimaging techniques have been extensively utilized to investigate brain structure and function across diverse populations, including healthy individuals, patients with neurological disorders, traumatic brain injury (TBI), and psychiatric conditions (Lee et al., 2013; Whitwell, 2009). While numerous studies have examined post-treatment structural and functional alterations in adult brain tumor patients (Xu et al., 2017; Almairac et al., 2018; Liu et al., 2020), research focusing on pediatric populations remains comparatively limited. Existing pediatric studies have predominantly concentrated on posterior fossa lesions and patients who underwent multimodal treatment (surgery, radiotherapy, and chemotherapy), leaving a notable gap in understanding the isolated effects of surgical intervention (Dennis et al., 2017; Leung et al., 2004; Jayakar et al., 2015).

Our previous investigations explored the impact of isolated surgical intervention on pediatric brain networks and cognitive function (Guan et al., 2022), including the compensatory responses of the contralateral hemisphere (Guan et al., 2023). However, longitudinal studies examining post-craniotomy cognitive, structural, and functional changes in pediatric neurosurgical patients remain scarce, resulting in a significant knowledge deficit in this domain. Although Zheng et al. (2023) investigated longitudinal changes in cognition and brain networks in children with frontal lobe lesions, their study was limited by a small sample size and restricted anatomical focus, preventing broader generalization to ISOLs as a comprehensive disease category.

The present study addresses this knowledge gap by investigating longitudinal changes in cognition, brain structure, and function within the contralateral hemisphere following isolated surgical treatment in 19 pediatric ISOL patients. Our findings potentially offer valuable insights into neuroplasticity and neurorehabilitation, while providing direction for future research endeavors.

## 2 Materials and methods

### 2.1 Subjects

We enrolled a total of 20 patients who were diagnosed with ISOLs and treated at the Department of Pediatric Neurosurgery, Beijing Tiantan Hospital between February 2021 and July 2023. One pediatric patient was excluded due to non-compliance with follow-up procedures, according to our inclusion and exclusion criteria detailed below, resulting in a final cohort of 19 participants.

Each patient underwent preoperative (Pre), first postoperative (Psot1), and second postoperative (Post2) MRI scans, as well as cognitive assessments.

The inclusion and exclusion criteria for the study are as follows:

Inclusion criteria: (a) Age: 6–18 years. (b) Disease: Primary ISOLs.
Exclusion criteria: (a) Children were diagnosed with hydrocephalus (Evan index >0.3). (b) The children are unable to finish the rs-fMRI scan successfully for any reasons, for example, children or their parents are unwilling to cooperate, or the head moves too much during the scanning, etc. (c) The patients had a history of craniocerebral trauma, craniocerebral surgery, mental and psychological diseases, genetic metabolic diseases, endocrine system diseases, etc.

Additionally, we recruited 10 healthy children from Beijing RIMAG Medical Imaging Center as a control group, matched for age and gender with no statistically significant differences from the patient group. The imaging equipment, acquisition parameters, and operating systems at Beijing RIMAG Medical Imaging Center were identical to those at Beijing Tiantan Hospital.

### 2.2 Ethical statements

Written informed consent was obtained from the parents of all enrolled pediatric patients. This prospective study adhered to the principles of the Declaration of Helsinki and received approval from the Institutional Review Board of Beijing Tiantan Hospital, Capital Medical University (KY 2021-100-02).

### 2.3 Cognitive assessment

All participant cognitive functions were assessed using the CNS Vital Signs (CNS VS) battery, a widely used computerized tool for clinical neurocognitive screening (Gualtieri and Johnson, 2006). The CNS VS battery is highly valid and reliable, with features that reduce practice effects in repeated measurements (Gualtieri and Johnson, 2006). This assessment takes about 30–40 min and generates standardized, age-adjusted scores across 15 cognitive domains based on 10 subtests. The domains evaluated include composite memory (CM), verbal memory (VerbM), visual memory (VisM), psychomotor speed (PsyMotSpd), reaction time (RT), complex attention (ComAtt), cognitive flexibility (CogFlex), processing speed (ProcSpd), executive function (ExeFun), social acuity (SocAcu), reasoning, working memory (WM), sustained attention (SustAtt), simple attention (SimAtt), and motor speed (MotSpd). The neurocognitive index (NCI), a summary score representing overall cognitive function, is derived from CM, PsyMotSpd, RT, CogFlex, and ComAtt. CNS VS scores have a normative mean of 100 and a standard deviation of 15. Each enrolled patient underwent three cognitive assessments, conducted at the Pre stage, Post1, and Post2. These evaluations were performed within the same week as the corresponding MRI scans.

### 2.4 Imaging data acquisition

The subjects were instructed to remain still with their eyes closed, without sedation during the examination. The imaging scan parameters were identical to those used in our previous study (Guan et al., 2022; Guan et al., 2023; Hu et al., 2024; Guan et al., 2024a,b). All patients underwent three MRI scans using the same 3 T scanner (MAGNETOM Prisma, Siemens Healthcare, Erlangen, Germany) equipped with a 64-channel head/neck coil. The protocol included T1 weighted structure imaging with magnetization prepared rapid acquisition gradient echo (MPRAGE) sequence and rs-fMRI with an echo-planar imaging (EPI) sequence. The scan parameters for the MPRAGE sequence were: repetition time (TR) = 1,560 ms; echo time (TE) = 1.65 ms; flip angle = 8°; slices = 176; field of view (FOV) = 256 × 256 mm; and voxel size = 1 mm isotropic. The parameters for EPI with simultaneous multislice (SMS) acceleration technique were: TR = 2000 ms; TE = 35 msec; slices = 69; SMS = 3; FOV = 207 × 207 mm; voxel size = 2.2 mm isotropic; volumes = 240.

### 2.5 Data preprocessing and analysis

Our study analyzed voxel-wised changes in structural and functional imaging of the contralesional hemisphere over long-term follow-up. For convenience, following previous study (Guan et al., 2023), we flipped the images from the three scans of the three patients with lesions located on the left side. Subsequently, the data from the three scans of all enrolled patients were preprocessed separately. Several structural and functional imaging metrics were then calculated to evaluate changes during the follow-up period. The detailed procedures are described below.

#### 2.5.1 Structural data

The processing and analysis of voxel-based morphometry (VBM) were conducted using Computational Anatomy Toolbox 12 (CAT12, version 12.7)^1^ and Statistical Parametric Mapping 12 (SPM12; version 7771),^2^ developed by the Wellcome Centre for Human Neuroimaging at the Institute of Neurology, University College London, and implemented in MATLAB R2020b (MathWorks, Inc.).

For image processing, the longitudinal model in CAT12, optimized for detecting subtle changes, was employed. The preprocessing pipeline included bias-field correction, noise reduction, skull stripping, and segmentation into gray matter (GM), white matter, and cerebrospinal fluid (CSF). Tissue segmentation was performed using the unified segmentation algorithm (Ashburner and Friston, 2005). Since the participants were children and adolescents, a child-specific tissue probability map was applied (Zhao et al., 2019).

Next, all GM images were normalized to the standard Montreal Neurological Institute (MNI) template using the diffeomorphic anatomical registration through exponential Lie algebra (DARTEL) method (Ashburner, 2007). This normalization utilized a 1.5 mm isotropic template provided by the CAT12 toolbox. Finally, normalized GM images underwent spatial smoothing with an 8 mm full width at half maximum (FWHM) Gaussian kernel.

#### 2.5.2 Functional data

The preprocessing of fMRI data was conducted using Data Processing and Analysis of Brain Imaging (DPABI) version 6.2^3^ and SPM12,^4^ both implemented in MATLAB R2020b (MathWorks, Inc.) (Yan et al., 2016; Chao-Gan and Yu-Feng, 2010). The preprocessing pipeline included the following steps:

(1) The first 10 images of each fMRI scan were discarded. (2) Slice-timing correction and head motion correction were applied. Participants with head motion exceeding 3 mm in translation or 3° in rotation were excluded. (3) Functional images were normalized to the standard MNI space using the DARTEL algorithm (Ashburner, 2007), followed by resampling to a voxel size of 3 × 3 × 3 mm^3^. (4) Several covariates, including the linear trend, signals from CSF and white matter, and the Friston 24-parameter head motion model, were regressed out from the BOLD signal (Friston et al., 1996). Notably, global signal regression was not performed during this preprocessing step (Saad et al., 2012; He and Liu, 2012). (5) Spatial smoothing was applied to the images using a Gaussian kernel with a FWHM of 4 × 4 × 4 mm^3^. (6) Band-pass filtering was conducted, retaining frequencies in the range of 0.01–0.10 Hz. (7) Three functional separation indices—regional homogeneity (ReHo) (Zang et al., 2004), amplitude of low-frequency fluctuations (ALFF) (Zang et al., 2007), and fractional ALFF (fALFF) (Zou et al., 2008)—were calculated. ReHo was computed before smoothing, while ALFF and fALFF were computed prior to filtering. All three indices underwent Fisher-*Z* transformation before statistical analyses.

### 2.6 Exploratory correlation analysis

We conducted Pearson correlation analyses between age, lesion volume, follow-up interval, and the change scores in cognitive domains showing significant differences, as well as imaging metrics of brain regions exhibiting significant alterations. The lesion volume was calculated using the formula *V* = 4/3*π* × *a*/2 × *b*/2 × *c*/2 (*a* and *b* = maximum perpendicular diameters on the axial images; *c* = diameter in the coronal direction on the sagittal or coronal images) (Guan et al., 2022; Guan et al., 2023; Osawa et al., 2013), which aims to more closely resemble the clinical estimation of lesion volume. To enhance the interpretability of the results, we specifically examined the correlations between changes in cognitive domains and their corresponding follow-up imaging metrics.

### 2.7 Statistical analysis and visualization

This study utilized a paired design to investigate whether various metrics showed statistically significant changes between Post1 and Pre, Post2 and Post1, as well as Post2 and Pre. To ensure the overall significance level of the study did not exceed 0.05, the significance threshold for individual comparisons was set at 0.016.

For imaging data, paired *t*-tests were conducted using the statistical module in DPABI. The significance threshold for individual comparisons was set at 0.016 to maintain an overall significance level below 0.05. During each comparison, imaging metrics were subjected to multiple comparison corrections. Specifically, we applied Gaussian random field (GRF) correction (voxel-level *p* < 0.001, cluster-level *p* < 0.016) provided by the DPABI statistical module.

For cognitive test data, paired *t*-tests were performed using SPSS 26, with the significance threshold for each test set at 0.016.

For correlation analyses, given their exploratory nature, we did not impose overly stringent significance thresholds, nor did we apply multiple comparison corrections. The significance level for these analyses was set at 0.05 (Guan et al., 2024a,b; Xu et al., 2022).

## 3 Results

### 3.1 Demographic and clinical characteristics

A total of 19 participants were included in the study, with 7 males (36.8%). The average age was approximately 10 years. The lesions in most of the enrolled patients were on the right side (14 cases, 73.7%), with one case located on the midline, and the remaining cases on the left side. The majority of the lesions were located in the frontal and temporal lobes (52.6%). The average lesion volume was 8.1 cubic centimeters. The most common initial symptom in the children was seizure (12 cases, 63.2%). Regarding the extent of resection, only one patient underwent a subtotal resection, while the others had a gross total resection (94.7%). The follow-up interval was calculated as the difference in days between the follow-up date and the surgery date. The average first follow-up interval was about 75 days, and the average second follow-up interval was about 316 days. Although the follow-up intervals were spread over a wide range, there was no overlap between the first and second follow-up periods. Detailed information can be found in Table 1.

**Table 1:** Demographic and clinical characteristics.

Item	Value
Age (mean ± SD, years)	10.0 ± 2.3 (range 6.6–15.2)
Gender (male:female)	7:12
Lesion lateralization (left:right:middle)	4:14:1
Lesion location (frontal:temporal:parietal:occipital:cerebellum)	6:4:5:2:2
Lesion volume^b^ (mean ± SD, cm^3^)	8.1 ± 11.6
Initial symptoms (seizure:asymptomatic:headache:intracranial hypertension)	12:3:2:2
Resection extent (gross total:subtotal)	18:1
Follow-up interval^a^ (first time) (mean ± SD, days)	74.8 ± 42.8
Follow-up interval^a^ (second time) (mean ± SD, days)	315.7 ± 137.8

a The time interval for the first follow-up ranged from 6 to 154 days, while the interval for the second follow-up was between 171 and 578 days. The follow-up intervals were non-overlapping.

b Lesion volume range: 0.1–37.5 cm^3^.

Regarding pathological diagnosis, this information is provided in the Supplementary Table 1. Our pathological diagnosis was based on the reports from the pathology department of our hospital, which referred to the 2016 classification of central nervous system tumors (Louis et al., 2016). All patients enrolled in the study had grade I lesions or non-tumor lesions.

### 3.2 Post-operative cognitive domains demonstrate initial decline followed by recovery trajectory

**Figure 1:**
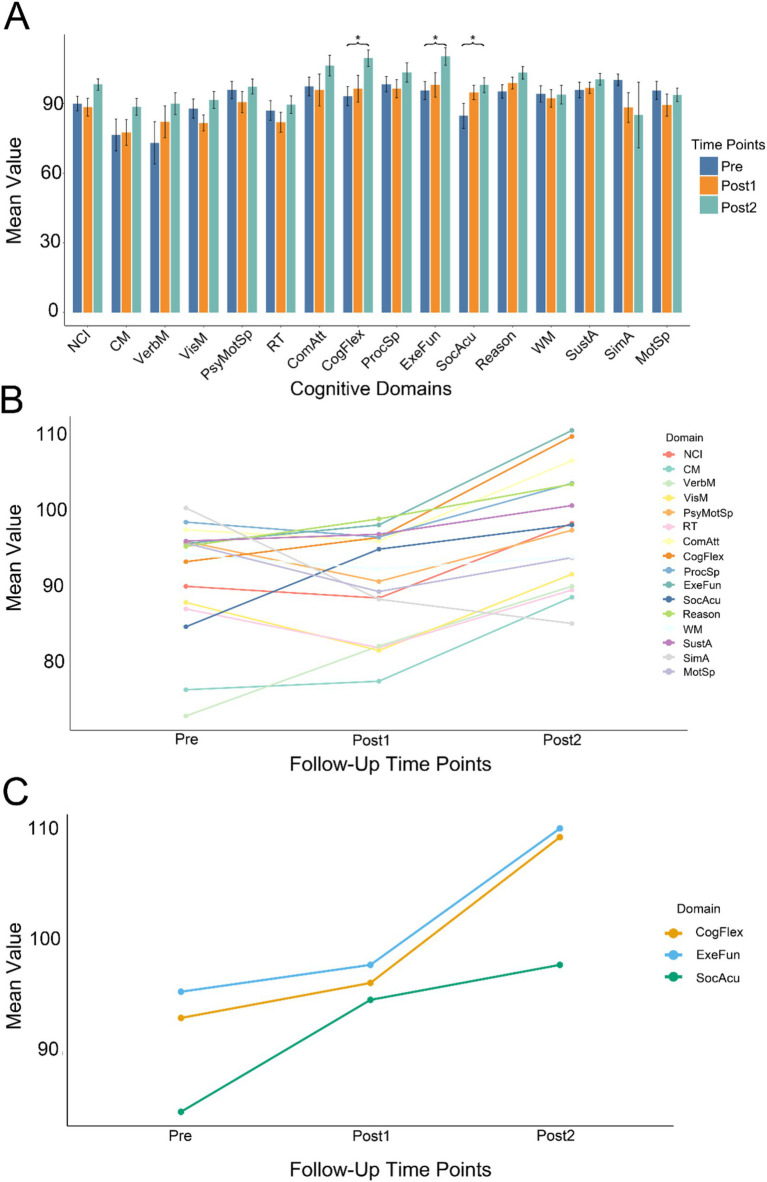
Longitudinal changes in cognitive function. **(A)** Summary of cognitive test results across three time points (Pre, Post1, and Post2) for all cognitive domains. Asterisks indicate statistical significance. Different colors represent different time points. Significant differences between Post2 and Pre were observed only in executive function (ExeFun), cognitive flexibility (CogFlex), and social acuity (SocAcu) domains (individual comparison significance level: 0.016; overall significance level: 0.05). **(B)** Temporal trends across all cognitive domains at three time points. Longitudinally, most cognitive domains demonstrated an upward trend. Some domains showed initial decline at Post1 followed by improvement at Post2. **(C)** Temporal progression of the three cognitive domains that exhibited significant changes.

For the cognitive assessments, we performed paired *t*-tests between Pre, Post1, and Post2. We found a significant difference only between Post2 and Pre, and this difference was observed exclusively in the CogFlex, ExeFun, and SocAcu domains. No significant differences were found in other domains. The results of the comparison between Post2 and Pre are presented in Table 2, while the results of the other two comparisons are provided in the Supplementary Tables 2, 3. Figure 1A shows bar charts for the three test times across the 16 cognitive domains, while Figure 1B displays line graphs illustrating the changes in these domains over time. Figure 1C highlights the temporal changes in the CogFlex, ExeFun, and SocAcu domains. From a general perspective, we observed that most cognitive domain scores declined to varying degrees at the first postoperative follow-up, but showed some recovery at the second follow-up. However, this recovery did not show significant differences when compared to the preoperative scores, except for the CogFlex, ExeFun, and SocAcu domains, which were significantly higher at the second follow-up compared to preoperative levels.

**Table 2:** Results of the comparison between preoperative (Pre) and second follow-up (Post2) cognitive testing^a^.

Test	Pre	Post2	*t*-value	*p*-value
NCI	89.95	98.16	2.444	0.025
CM	76.47	88.53	1.709	0.105
VerbM	73.05	89.95	1.880	0.076
Vism	87.84	91.53	0.758	0.458
PsyMotSp	95.79	97.26	0.424	0.677
RT	87.00	89.47	0.527	0.604
ComAtt	97.32	106.32	2.306	0.033
CogFlex^*^	93.16	109.47	4.201	0.001^*^
ProcSp	98.32	103.37	1.589	0.129
ExeFun^*^	95.53	110.26	3.478	0.003^*^
SocAcu^*^	84.68	97.95	3.248	0.004^*^
Reason	95.16	103.26	2.188	0.042
WM	94.11	93.79	−0.060	0.953
SustA	95.84	100.47	1.421	0.172
SimA	100.16	85.11	−1.089	0.291
MotSp	95.58	93.68	−0.515	0.613

The asterisks symbol denotes statistically significant differences. CM, composite memory; VerbM, verbal memory; VisM, visual memory; PsyMotSpd, psychomotor speed; RT, reaction time; ComAtt, complex attention; CogFlex, cognitive flexibility; ProcSpd, processing speed; ExeFun, executive function; SocAcu, social acuity; Reason, reasoning; WM, working memory; SustAtt, sustained attention; SimAtt, simple attention; MotSpd, motor speed; NCI, neurocognitive index.

a The significance level was set at 0.016. The *t*-value reflects the discrepancy between the second follow-up data and the preoperative data.

### 3.3 Structural alterations precede functional changes: a progression from subcortical to cortical regions

**Figure 2:**
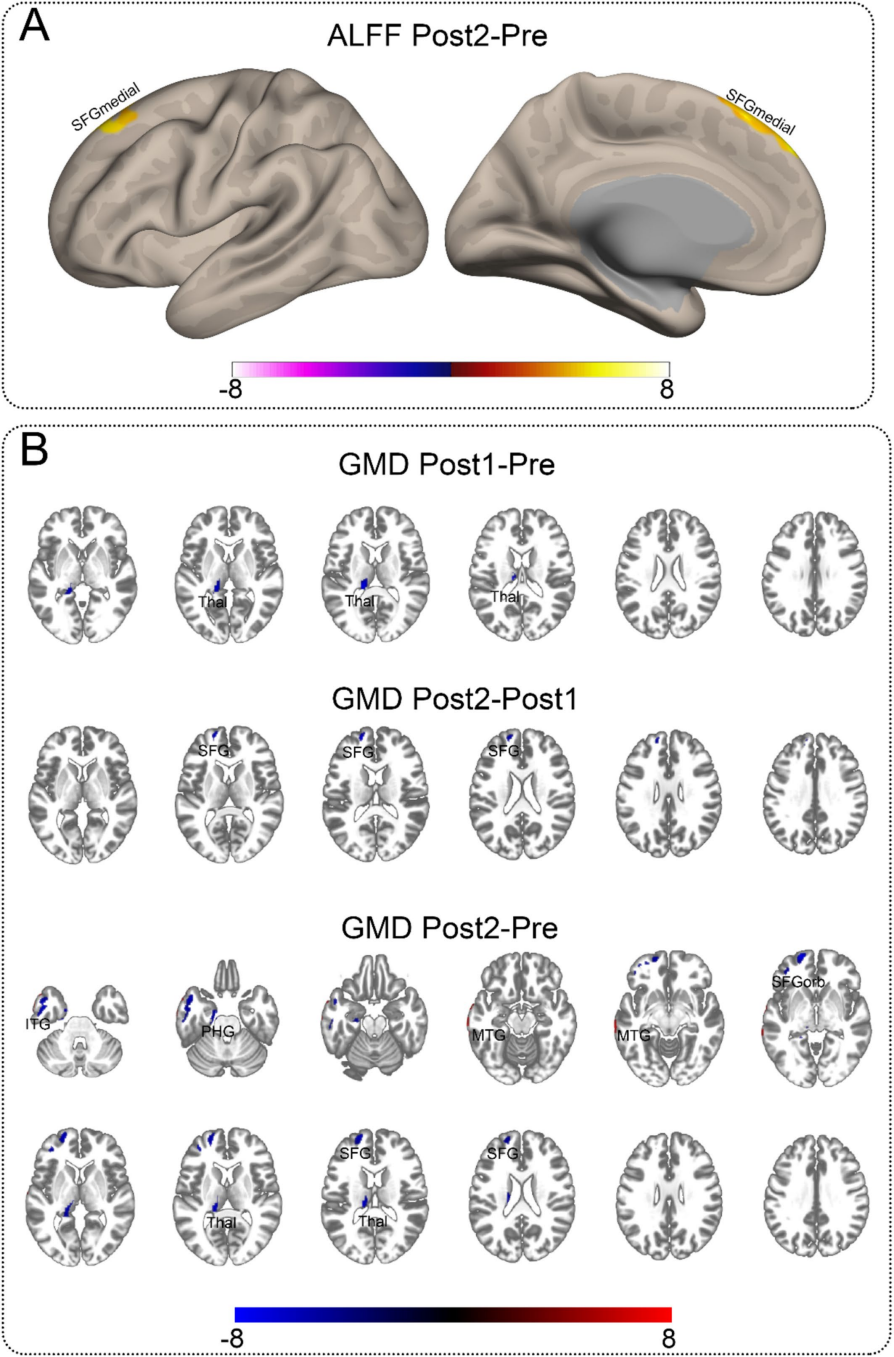
Significant neuroimaging findings in the contralateral hemisphere. This illustration depicts brain regions demonstrating significant differences. The abbreviations marked on the figure indicate the approximate locations of peak regions or areas with maximal proportional involvement, provided as reference points for readers. Detailed coordinates can be found in Table 3. **(A)** Significant differences in ALFF between Post2 and Pre time points. Among all functional metrics, only ALFF showed significant differences between Post2 and Pre, while other functional indicators showed no significant differences between any time points. **(B)** Gray matter density comparisons in the contralateral hemisphere. Significant differences were observed between all paired time points (Pre, Post1, and Post2). The comparison between Post1 and Pre revealed decreased gray matter density in subcortical structures. Between Post2 and Post1, cortical regions showed decreased gray matter density. The comparison between Post2 and Pre demonstrated gray matter density reduction in both cortical and subcortical regions, along with increased gray matter density in certain temporal lobe areas. ITG, inferior temporal gyrus; MTG, middle temporal gyrus; PHG, parahippocampal gyrus; SFG, superior frontal gyrus, dorsolateral; SFGmedial, superior frontal gyrus, medial; SFGorb, superior frontal gyrus, orbitalis; Thal, thalamus.

For the rs-fMRI data, we compared ReHo, ALFF, and fALFF. We found that only ALFF showed a significant difference between Post2 and Pre. For structural MRI, we compared gray matter density (GMD). We observed differences between Pre, Post1, and Post2 in pairs, with the distribution of significant brain regions primarily concentrated in the frontal and temporal lobes, as well as the thalamus. Most of the brain regions showing significant differences exhibited a decrease of GMD over time. However, the ALFF in the medial superior frontal gyrus showed a notable increase at the second postoperative follow-up compared to the preoperative levels. Additionally, the GMD in the middle temporal gyrus was higher at the second postoperative follow-up than before surgery. Detailed information can be found in Table 3. Figure 2 presents the visualized results. Figure 2A shows the fMRI results, while Figure 2B displays the statistical comparison results for GMD.

**Table 3:** The results of brain imaging analysis.

Item (contrast)	Cluster No.	Cluster size (voxels)	Peak coordinate (*X*, *Y*, *Z*)	Peak intensity	Peak label	Label with most voxels (%)
ALFF (Post2-Pre)						
	Cluster 1	47	−6, 33, 57	5.96	Frontal_Sup_Medial	Frontal_Sup_Medial (66.0%)
GMD (Post1-Pre)						
	Cluster 1	549	−15, −28.5, 15	−7.54	None	Thalamus (54.3%)
GMD (Post2-Post1)						
	Cluster 1	413	−18, 54, 13.5	−7.80	Frontal_Sup	Frontal_Sup (85.0%)
GMD (Post2-Pre)						
	Cluster 1	1,264	−21, 61.5, -1.5	−7.55	Frontal_Sup_Orb	Frontal_Sup (46.2%)
	Cluster 2	845	−45, −1.5, −36	−7.20	Temporal_Inf	Temporal_Inf (64.4%)
	Cluster 3	677	−69, −25.5, −10.5	7.57	Temporal_Mid	Temporal_Mid (60.9%)
	Cluster 4	627	−19.5, −18, −30	−6.01	None	ParaHippocampal (20.7%)
	Cluster 5	618	−15, −24, 15	−6.15	Thalamus	Thalamus (58.3%)

ALFF, amplitude of low-frequency fluctuations; GMD, gray matter density; Frontal_Sup_Medial, superior frontal gyrus, medial; Frontal_Sup, superior frontal gyrus, dorsolateral; Frontal_Sup_Orb, superior frontal gyrus, medial orbital; Temporal_Inf, inferior temporal gyrus; Temporal_Mid, middle temporal gyrus.

Additionally, we conducted a two-sample *t*-test to compare the GMD of the unaffected hemisphere scanned three times with the GMD of the control group. The overall significance level was set at 0.05, with the significance level for each comparison set at 0.016. We found no differences between the Pre and the control and Post2 and the control; however, a significant difference was observed only between Post1 and the control group, specifically in the cerebellum. The statistically significant results from this comparison with the control group are presented in the Supplementary Table 4.

### 3.4 Correlations among cognitive function, follow-up duration, and structural brain changes

**Figure 3:**
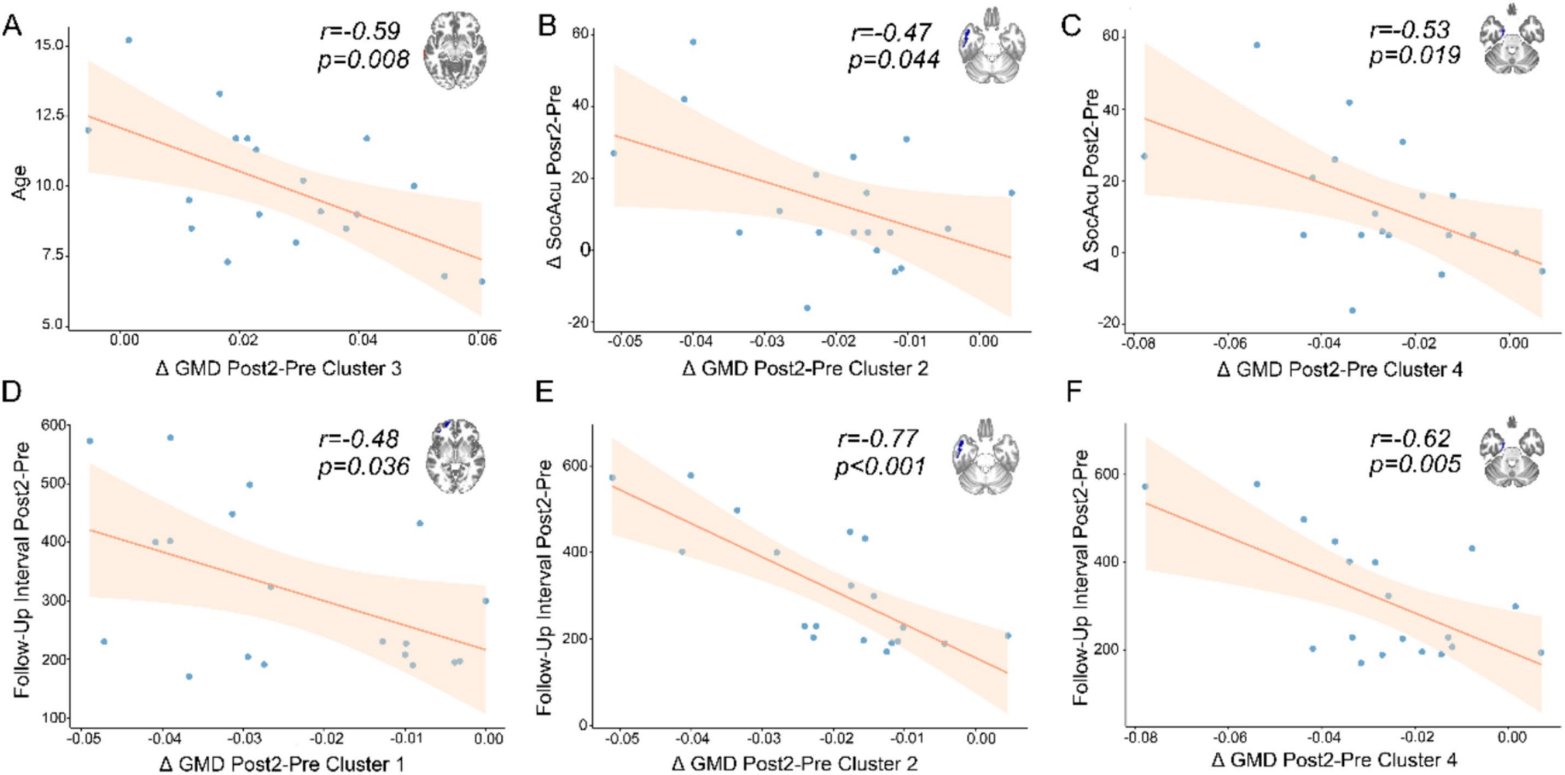
The results of correlation analysis. The *x*-axes represent the mean differences between Post2 and Pre timepoints for clusters showing significant changes. The *y*-axes indicate different metrics. **(A)** Negative correlation between age and the difference in gray matter density (GMD) in the middle temporal gyrus. **(B)** Negative correlation between changes in social acuity (SocAcu) and differences in the inferior temporal gyrus. **(C)** Negative correlation between changes in social acuity and differences in the parahippocampal gyrus. **(D–F)** Negative correlations between follow-up intervals (Post2 vs. Pre) and differences in the orbital part of the superior frontal gyrus, inferior temporal gyrus, and parahippocampal gyrus, respectively.

We conducted Pearson correlation analysis between the differences in imaging indices with significant changes, corresponding significant cognitive domain score differences, and demographic information. We found that only the difference between the second follow-up and preoperative measurements showed significant correlations with certain indices (Figure 3). We discovered a negative correlation between the age of the child at surgery and the increased GMD in the middle temporal gyrus (Figure 3A). Additionally, improvements in social cognition were found to correlate with a decrease in the GMD in certain temporal lobe structures (Figures 3B,C). The time interval between the second follow-up and surgery was correlated with a decrease in the GMD in some structures of the frontal and temporal lobes. Based on our current data, the reduction in GMD appears to become more pronounced with increasing follow-up intervals (Figures 3E–G). However, due to the absence of extended longitudinal follow-up data, we cannot definitively determine whether this trend would persist over a longer time course.

## 4 Discussion

This study fills the gap in research on cognitive function and the longitudinal changes in brain structure and function following pediatric neurosurgical procedures. We found that after pediatric craniotomy, the healthy hemisphere underwent structural changes from subcortical to cortical areas, a process that is slow and cumulative in nature. More importantly, structural changes generally precede functional changes. These findings may provide new insights into the structural and functional basis underlying the post-operative improvement of cognitive function in children, offering new clues for understanding the related neuroplasticity mechanisms.

Based on our results, most cognitive function improved post-surgery longitudinally, although significant differences were only observed between Post2 and Pre in the domains of SocAcu, CogFlex, and ExeFun. The results of the correlation analysis indicate that only SocAcu is associated with changes in brain structure. However, previous studies suggest that changes in higher cognitive functions involve multiple brain regions (Stuss, 2011; Kim et al., 2011; Chen et al., 2014; Liu et al., 2021; Li et al., 2013). Therefore, despite the absence of linear correlations between some cognitive functions and brain region metrics, these functions may still be influenced by structural and functional changes in these regions.

According to brain imaging analyses, we found that the earliest detectable structural alteration occurred in the thalamus, a subcortical structure, specifically in its GMD. At the first postoperative follow-up (approximately 2.5 months after surgery), a reduction in thalamic GMD was already evident, while functional imaging metrics did not show statistically significant differences at this time point. By the second follow-up (approximately 10.5 months postoperatively), compared to the first follow-up, thalamic GMD did not exhibit significant changes; however, a reduction in GMD was observed in the frontal lobe, a cortical structure, while functional imaging metrics still did not show statistically significant differences. When comparing the second follow-up to the preoperative baseline, we observed many changes in both cortical and subcortical structures, predominantly characterized by reductions in GMD. Notably, at this time point, ALFF in the frontal lobe significantly increased compared to preoperative levels, suggesting enhanced functional activity in this region. Further correlation analyses revealed that improvements in the SocAcu metric were associated with reductions in GMD, suggesting that this decline in GMD may represent a potential compensatory mechanism following pediatric brain surgery.

Previous studies have reported similar findings. For instance, Catharine et al. (2019) investigated the effects of executive function-related cognitive training in children with moderate-to-severe TBI. The authors reported a negative correlation between changes in the performance of the Digit Symbol Substitution Test and putamen volume, indicating that reductions in putamen volume were associated with improved test performance. This finding aligns, to some extent, with our results, as reductions in GMD/volume appear to be associated with cognitive improvement.

Similarly, Moberget et al. (2015) examined supratentorial brain structures and cognitive function in adult patients who had undergone treatment for childhood cerebellar tumors, comparing them with matched controls. They found that, compared to the control group, patients exhibited cognitive decline and increased GMD in the bilateral cingulate cortex, left orbitofrontal cortex, and left hippocampus. Furthermore, in the patient group, increased GMD in these regions was negatively correlated with performance on processing speed and executive function tasks. This study provides indirect support for our results, further suggesting that reductions in GMD may be linked to cognitive improvement from an alternative perspective.

Dennis et al. (2017) offer additional evidence supporting this notion. In their longitudinal study on regional brain volume changes following pediatric TBI, they found that children with TBI exhibited reductions in GM volume, which were also correlated with improvements in cognitive abilities. Dennis et al. (2017) proposed that since reductions in GM volume are consistent with normal developmental expectations, these changes may represent signs of returning to a healthy developmental trajectory. During normal childhood development, GMD reductions occur, potentially driven by synaptic pruning, a process that enhances neural efficiency and improve cognitive capacity (Gogtay et al., 2004; Dennis and Thompson, 2013; Sowell et al., 2001).

Our findings appear to resemble the synaptic pruning observed during typical neurodevelopment, in which reductions in GMD are accompanied by cognitive improvements. Previous studies in healthy adults have demonstrated that training-induced neuroplasticity may parallel developmental plasticity (Thomas and Baker, 2013; Wenger et al., 2017), characterized by initial GM volume expansion (attributed to neurogenesis, glial cell proliferation, dendritic spine growth, and synaptogenesis), followed by gradual reduction through dendritic and synaptic pruning (Zhou et al., 2015; Treit et al., 2014; Maxwell, 2012). Moreover, several investigators have proposed that alterations in MRI signals may reflect changes in axonal myelination, neurogenesis, angiogenesis, dendritic spine dynamics, glial cell proliferation, and synaptogenesis (Scholz et al., 2009; Draganski and May, 2008). Synthesizing these previous investigations suggests that both injury-induced and training-induced neuroplasticity share associations with synaptic pruning mechanisms. This convergence lends support to our observed findings.

Given the aforementioned studies and our current findings, we cautiously propose that the restoration of structural and functional integrity in the contralateral hemisphere following pediatric neurosurgery follows a trajectory from subcortical to cortical regions. Structural alterations, particularly GMD reduction, appear to precede functional changes. This recovery mechanism may be driven by an externally induced “pruning-like” process, triggered by surgical intervention or other external perturbations, facilitating its activation to assist neural repair. The term “pruning-like” is employed because our mesoscopic-level neuroimaging study precludes definitive mechanistic determination, allowing only inference based on previous investigations. Our comparisons between patients and normally developing children revealed no significant differences in cortical and subcortical GMD, and our overall follow-up period of approximately 1 year is relatively brief. These observations suggest that the GMD changes observed in our cohort may not be developmentally driven but rather injury-induced. Such injury-driven “pruning-like” modifications have been observed in animal studies. Campbell et al. (2012) discovered that rats subjected to fluid percussion injury exhibited reduced total spine density at 24 h post-injury, suggesting that this reduction might be associated with synaptic dynamics.

While this “plasticity” should theoretically facilitate cognitive rehabilitation in pediatric patients, numerous studies indicate that “plasticity” is not invariably beneficial (Anderson et al., 2011). For instance, atypical pruning may lead to various neurodevelopmental disorders, including schizophrenia, autism spectrum disorders, and epilepsy (Neniskyte and Gross, 2017). Notably regarding epilepsy, both central nervous system tumors and neurosurgical interventions can precipitate seizure disorders. Mofatteh et al. (2024) summarized multiple explanatory mechanisms, including chronic subcortical network injury and denervation supersensitivity, as potential pathophysiological mechanisms underlying seizure generation. The pruning-like mechanism appears to integrate well with these mechanisms: atypical synaptic pruning induced by chronic subcortical network injury leading to epileptogenesis. Mofatteh et al. (2024) further noted that some studies demonstrate the ineffectiveness of prophylactic antiepileptic drugs in preventing postoperative epilepsy, while other case reports suggest the feasibility of aggressive perioperative prophylactic antiepileptic medication. The review by Ikonomidou and Turski (2010) indicates that antiepileptic drugs significantly impact synaptogenesis and remodeling, affecting synaptic pruning. According to our pruning-like hypothesis, this may partially explain the heterogeneous response to antiepileptic medications, as their administration likely influences synaptic pruning mechanisms, thereby altering subsequent developmental trajectories.

Our correlation analyses suggest that GMD reduction becomes more pronounced with extended follow-up periods and may potentially persist for a duration. If surgical injury indeed induces pruning-like mechanisms, and if this pruning process becomes aberrant—whether through prolonged duration or manifestation of “atypical” or “non-canonical” patterns—it might ultimately result in cognitive decline among affected children. Thus, our hypothesis regarding this pruning-like mechanism may help explain why some children demonstrate suboptimal recovery trajectories despite substantial evidence supporting pediatric brain plasticity. However, due to study design limitations, we can currently only confirm what our data demonstrates: a correlation between GMD and cognitive improvement. The longer-term outcomes remain difficult to ascertain and warrant further investigation.

There are several limitations of our study. Primarily, from a methodological perspective, the follow-up duration constrains our result interpretation. The absence of extended longitudinal follow-up precludes observation of future cognitive changes and neuroimaging alterations in pediatric patients. Additionally, the sample size limits our ability to draw broader conclusions. Future investigations should incorporate larger cohorts and extend the longitudinal follow-up period to establish more comprehensive temporal dynamics. We did not conduct comparisons of functional imaging data between enrolled patients and the control group; this represents an area for improvement in future investigations.

## 5 Conclusion

Our study suggests that post-operative neural recovery following ISOLs in children follows a self-repair trajectory, which appears to progress from subcortical to cortical regions. This trajectory is characterized by a structural reduction in GMD, followed by functional activation. Moreover, this phenomenon exhibits slow and cumulative characteristics. The potential underlying neuroplastic mechanisms may involve external disturbances that induce a synaptic-pruning-like process. Cognitive function demonstrated improvement during the mean follow-up period of approximately 1 year; however, longer-term changes warrant continued vigilant monitoring. Our findings offer new insights for future research on the mechanisms of self-repair following brain injury during development, providing valuable perspectives for the fields of neuroplasticity and neurorehabilitation, while highlighting the necessity for extended longitudinal monitoring of post-operative cognitive functional changes.

## Data Availability

The raw data supporting the conclusions of this article will be made available by the authors, without undue reservation.

## Footnotes

^1^ http://www.neuro.uni-jena.de/cat/

^2^ https://www.fil.ion.ucl.ac.uk/spm

^3^ http://rfmri.org/dpabi

^4^ https://www.fil.ion.ucl.ac.uk/spm

## Glossary

ALFF: Amplitude of low frequency fluctuations
BOLD: Blood oxygen-level-dependent
CAT: Computational anatomy toolbox
CM: Composite memory
CNS VS: CNS vital signs
CogFlex: Cognitive flexibility
ComA: Complex attention
DARTEL: Diffeomorphic anatomical registration through exponential Lie algebra
DPABI: Data Processing and Analysis of Brain Imaging
EPI: Echo-planar imaging
ExeFun: Executive function
fALFF: Fractional ALFF
FOV: Field of view
FWHM: Full width at half maximum
GM: Gray matter
GMD: Gray matter density
GRF: Gaussian random-field theory
ISOLs: Intracranial space-occupying lesions
MNI: Montreal Neurological Institute
MotSpd: Motor speed
MPRAGE: Magnetization prepared rapid acquisition gradient echo
NCI: Neurocognitive index
ProcSpd: Processing speed
PsyMotSpd: Psychomotor speed
Reason: Reasoning
ReHo: Regional homogeneity
rs-fMRI: Resting-state functional magnetic resonance imaging
RT: Reaction time
SimA: Simple attention
SMS: Simultaneous multislice
SocAcu: Social acuity
SPM: Statistical parametric mapping
SustA: Sustained attention
TBI: Traumatic brain injury
TE: Echo time
TR: Repetition time
VBM: Voxel-based morphometry
VerbM: Verbal memory
VisM: Visual memory
WM: Working memory
