# Supplementary material for: Structural and functional alterations in the contralateral hemisphere following pediatric intracranial surgery: a pilot longitudinal neuroimaging study
Source: Front Hum Neurosci. 2025 Mar 7;19:1568945. doi: 10.3389/fnhum.2025.1568945 (PMC11925946; doi:10.3389/fnhum.2025.1568945)
Supplement: Supplementary file 1 [file Table_1.docx]

Supplementary Table 1 Pathological Diagnosis

| ID | Diagnosis | WHO grade |
| --- | --- | --- |
| sub001 | Dysembryoplastic neuroepithelial tumor | Ⅰ |
| sub002 | Ganglioglioma | Ⅰ |
| sub003 | Dysembryoplastic neuroepithelial tumor | Ⅰ |
| sub004 | Epidermoid cyst |  |
| sub005 | Cavernous malformation |  |
| sub006 | Neuronal and mixed neuronal-glial tumors | Ⅰ |
| sub007 | Cavernous malformation |  |
| sub008 | Cavernous malformation |  |
| sub009 | Angiocentric glioma | Ⅰ |
| sub010 | Dysembryoplastic neuroepithelial tumor | Ⅰ |
| sub011 | Cavernous malformation |  |
| sub012 | Ganglioglioma | Ⅰ |
| sub013 | Ganglioglioma | Ⅰ |
| sub014 | Cavernous malformation |  |
| sub015 | Neuronal and mixed neuronal-glial tumors | Ⅰ |
| sub016 | pilocytic astrocytoma | Ⅰ |
| sub017 | Ganglioglioma | Ⅰ |
| sub018 | Ganglioglioma | Ⅰ |
| sub019 | Neuronal and mixed neuronal-glial tumors | Ⅰ |

Supplementary Table 2 Results of the Comparison between Preoperative (Pre) and First Follow-up (Post1) Cognitive Testing^&^

|  | Pre | Post 1 | T-value | P-value |
| --- | --- | --- | --- | --- |
| NCI | 89.95 | 88.42 | -0.357 | 0.725 |
| CM | 76.47 | 77.58 | 0.151 | 0.882 |
| VerbM | 73.05 | 82.16 | 0.975 | 0.343 |
| Vism | 87.84 | 81.58 | -1.465 | 0.160 |
| PsyMotSp | 95.79 | 90.58 | -1.412 | 0.175 |
| RT | 87.00 | 81.95 | -1.097 | 0.287 |
| ComAtt | 97.32 | 95.84 | -0.252 | 0.804 |
| CogFlex | 93.16 | 96.32 | 0.564 | 0.580 |
| ProcSp | 98.32 | 96.37 | -0.590 | 0.562 |
| ExeFun | 95.53 | 97.95 | 0.425 | 0.676 |
| SocAcu | 84.68 | 94.79 | 1.950 | 0.067 |
| Reason | 95.16 | 98.74 | 1.185 | 0.252 |
| WM | 94.11 | 92.21 | -0.548 | 0.591 |
| SustA | 95.84 | 96.74 | 0.368 | 0.717 |
| SimA | 100.16 | 88.26 | -1.984 | 0.063 |
| MotSp | 95.58 | 89.26 | -1.737 | 0.099 |

& The significance level was set at 0.016. The t-value reflects the discrepancy between the first follow-up data and the preoperative data

Supplementary Table 3 Results of the Comparison between First Follow-up (Post1) and Second Follow-up (Post2) Cognitive Testing^&^

|  | Post 1 | Post 2 | T-value | P-value |
| --- | --- | --- | --- | --- |
| NCI | 88.42 | 98.16 | 2.330 | 0.032 |
| CM | 77.58 | 88.53 | 1.579 | 0.132 |
| VerbM | 82.16 | 89.95 | 0.878 | 0.392 |
| Vism | 81.58 | 91.53 | 2.279 | 0.035 |
| PsyMotSp | 90.58 | 97.26 | 2.027 | 0.058 |
| RT | 81.95 | 89.47 | 1.692 | 0.108 |
| ComAtt | 95.84 | 106.32 | 1.498 | 0.151 |
| CogFlex | 96.32 | 109.47 | 2.315 | 0.033 |
| ProcSp | 96.37 | 103.37 | 1.750 | 0.097 |
| ExeFun | 97.95 | 110.26 | 2.334 | 0.031 |
| SocAcu | 94.79 | 97.95 | 0.739 | 0.469 |
| Reason | 98.74 | 103.26 | 1.929 | 0.070 |
| WM | 92.21 | 93.79 | 0.298 | 0.769 |
| SustA | 96.74 | 100.47 | 1.239 | 0.231 |
| SimA | 88.26 | 85.11 | -0.203 | 0.841 |
| MotSp | 89.26 | 93.68 | 1.410 | 0.176 |

& The significance level was set at 0.016. The t-value reflects the discrepancy between the second follow-up data and the first follow-up data

Supplementary Table 4 The results of GMD between Post1 and the control group

| Item (Contrast) | Cluster No. | Cluster Size (Voxels) | Peak Coordinate (X,Y,Z) | Peak Intensity | Peak Label | Label with most voxels (%) |
| --- | --- | --- | --- | --- | --- | --- |
| GMD (Post1-Pre) |  |  |  |  |  |  |
|  | Cluster 1 | 1285 | -15,-91.5,28.5 | -6.41 | Cerebelum_Crus2_L | Cerebelum_Crus2_L (75.5%) |
